# Evaluation of the Phytochemical, Antioxidant, Enzyme Inhibition, and Wound Healing Potential of *Calotropis gigantea* (L.) Dryand: A Source of a Bioactive Medicinal Product

**DOI:** 10.3389/fphar.2021.701369

**Published:** 2021-08-17

**Authors:** Ahmed Alafnan, Swathi Sridharagatta, Hammad Saleem, Umair Khurshid, Abdulwahab Alamri, Shabana Yasmeen Ansari, Syafiq Asnawi Zainal Abidin, Siddique Akber Ansari, Abdulhakeem S. Alamri, Nafees Ahemad, Sirajudheen Anwar

**Affiliations:** ^1^ Department of Pharmacology and Toxicology, College of Pharmacy, University of Hail, Hail, Saudi Arabia; ^2^ Department of Pharmacognosy, Vydehi Institute of Pharmacy, Bangalore, India; ^3^ Institute of Pharmaceutical Sciences (IPS), University of Veterinary and Animal Sciences (UVAS), Lahore, Pakistan; ^4^ School of Pharmacy, Monash University Malaysia, Darul Ehsan, Malaysia; ^5^ Bahawalpur College of Pharmacy, Bahawalpur Medical and Dental College, Bahawalpur, Pakistan; ^6^ Department of Chemical and Electronic Engineering, Pharmaceutical Unit, University of Messina, Messina, Italy; ^7^ Liquid Chromatography Mass Spectrometry (LCMS) Platform, Jeffrey Cheah School of Medicine and Health Sciences, Monash University Malaysia, Selangor Darul Ehsan, Malaysia; ^8^ Department of Pharmaceutical Chemistry, College of Pharmacy, King Saud University, Riyadh, Saudi Arabia; ^9^ Department of Clinical Laboratory Sciences, College of Applied Medical Science, Taif University, Taif, Saudi Arabia

**Keywords:** *Calotropis gigantea*, phytochemicals, phenolic content, antioxidant, enzyme inhibition, wound healing

## Abstract

Traditionally, plants of the genus *Calotropis* have been used to cure various common diseases. The present research work explores the chemical and biological characterization of one of the most common species of this genus, i.e., *Calotropis gigantea* (L.) Dryand (syn. *Calotropis gigantea* (L.) Dryand.), having multiple folklore applications. The ethanolic extract of leaves of *Calotropis gigantea* (L.) Dryand was analyzed for the phytochemical composition by determining the total bioactive (total phenolic and total flavonoid) contents and UHPLC-MS secondary metabolites analysis. For phytopharmacological evaluation, *in vitro* antioxidant (including DPPH, ABTS, FRAP, CUPRAC, phosphomolybdenum, and metal chelation antioxidant assays) activities, enzyme inhibition potential (against AChE, BChE, α-amylase, and tyrosinase enzymes), and *in vivo* wound healing potential were determined. The tested extract has been shown to contain considerable flavonoid (46.75 mg RE/g extract) and phenolic (33.71 mg GAE/g extract) contents. The plant extract presented considerable antioxidant potential, being the most active for CUPRAC assays. Secondary metabolite UHPLC-MS characterization, in both the positive and negative ionization modes, indicated the tentative presence of 17 different phytocompounds, mostly derivatives of sesquiterpene, alkaloids, and flavonoids. Similarly, the tested extract exhibited considerable inhibitory effects on tyrosinase (81.72 mg KAE/g extract), whereas it showed weak inhibition ability against other tested enzymes. Moreover, in the case of *in vivo* wound healing assays, significant improvement in wound healing was observed in both the tested models at the doses of 0.5 percent w/w (*p < 0.001*) and 2.0 percent w/w (*p < 0.01*) on the 16^th^ day. The outcomes of the present research work suggested that *C. gigantea* (L.) Dryand plant extract could be appraised as a potential origin of bioactive molecules having multifunctional medicinal uses.

## Introduction

The kingdom of plants is a source of a vast spectrum of medicinally active compounds. Plants have synthesized these compounds using different metabolic pathways. Therefore, it is essential to establish a quick and reliable method for screening plant extracts (Cuthbertson et al., 2013). Plants, with a huge number of bioactive ingredients, are the primary suppliers of natural herbal medicines that are effective against different diseases, including cancer, and these bioactive components are associated with the biological activities of plants (Ahmad et al., 2016; Mahomoodally et al., 2019). Over the last few decades, the importance of herbal medicines for the treatment of various diseases has risen exponentially because a vast number of people belonging to diverse cultures rely on the use of phytomedicines due to a shortage of primary health facilities (Shanmugam and Bhavani, 2014). According to the World Health Organization reports on phytomedicines, more than 25 percent of the drugs prescribed in recent years come from various plant sources (Khurm et al., 2016). Interestingly, due to the beneficial health effects of medicinal herbs, herbal pharmaceutical manufacturing has evolved into an ever-growing industry (Suroowan and Mahomoodally, 2019). Accordingly, there is an increased interest in exploring the folklore and conventionally used medicinal flora to discover new bioactive compounds (Aumeeruddy and Mahomoodally, 2019).

The *Calotropis gigantea* (L.) Dryand (*C. gigantea*) or sweet akand of the Asclepiadaceae family is native to India and grows well at 900 m in the lower hills (Ghule et al., 2014). Various parts of *C. gigantea* (L.) Dryand are documented for the treatment of sprain, agitation, fatigue, epilepsy, mental conditions, diarrhea, analgesic intervention, and the interceptive properties of pregnancy, toothache, and earache (Argal and Pathak, 2006; Pathak and Argal, 2007; Ghule et al., 2014). Regarding the secondary metabolites, the different species of the *Calotropis* genus have been reported for the presence of several important classes of phytochemicals, including glycosides, flavonoids, fatty acids, and triterpenoids (Srivastava et al., 2015).

Taking into consideration the dietary and herbal uses of this plant as reported earlier, the current research plans to evaluate the detailed phytochemical composition (total phenolic and flavonoid content, ultra-high-performance liquid chromatography-mass spectrometry (UHPLC-MS), secondary metabolites characterization) and conduct *in vitro* and *in vivo* biological evaluation (antioxidant, enzyme inhibition, and wound healing activities) of *C. gigantea* (L.) Dryand leaves ethanolic extract. Antioxidant potential was appraised by testing free radical scavenging activity (DPPH and ABTS), reducing power (FRAP, ferric reducing antioxidant power; CUPRAC, cupric reducing antioxidant power), phosphomolybdenum total antioxidant capacity, and ferrous ion chelation (metal chelation) assays. Similarly, the capacity for inhibition against clinically important enzymes involved in major pathologies, including neurological disorders (acetylcholinesterase, AChE; butyrylcholinesterase, BChE), skin conditions (tyrosinase), and diabetes (α-amylase), were also examined. Additionally, *in vivo* wound healing activity determined by excision wound and burn wound models were also assessed.

## Material and Methods

### Plant Collection and Extraction

*Calotropis gigantea* (L.) Dryand (syn. *Calotropis gigantea* (L.) Dryand.) leaves were obtained and authenticated from Leaf Cleantech Private Limited. Approximately 50 g of powdered leaves with 400 ml of ethanol was extracted for 8 h using the Soxhlet unit. The resultant extract was concentrated and evaporated into dryness at a low temperature by distillation. Then, the extract was weighed, and the extractive percentage was measured in terms of the air-dried weight of the plant material.

### Animals

Seven-week-old male Wistar albino rats (180–200 g) purchased from Bioneeds, Nelamangala, Tumkur, were maintained in the animal house, maintained in a clean room with a light-dark cycle of 12/12, 18–26°C temperature, and 30–70 percent relative humidity. Rats are initially acclimatized for one week and served water ad libitum with a daily chow diet. In solid-bottom caging, rats were caged with bedding with enough room and washed regularly. In accordance with international guidelines, all laboratory experiments (Garber et al., 2011), including animal care and handling, feeding, and scarification, were approved by the institute (IAEC/2019). After the experiment, all the animals in the treated group were euthanized with excessive isoflurane and CO_2_ inhalation.

### Total Bioactive Contents

The total bioactive contents of *C. gigantea* (L.) Dryand leaves ethanolic extract were evaluated by analyzing total phenolic and flavonoid contents. Using a well-established Folin–Ciocalteu reagent process, the total phenolic content was estimated (Kähkönen et al., 1999), and the values were presented as milligrams of gallic acid equivalent per gram of extract (mg GAE/g extract). Similarly, total flavonoid content was measured using the colorimetric method of aluminum chloride (Chew et al., 2009), and the results were expressed as milligrams of rutin equivalent per gram of extract (mg RE/g extract).

### UHPLC-MS Phytochemical Analysis

After determining the total bioactive contents, the individual secondary metabolites profiling of *C. gigantea* (L.) Dryand leaves ethanolic extract was achieved via UHPLC-MS analysis in both the positive and negative ionization modes. Agilent Zorbax Eclipse XDB-C18 column with a narrow bore of 2.1 × 150 mm, 3.5 μm (P/N: 930990-902) was utilized. The temperature of the autosampler and column was kept at 4°C and 25°C, respectively. The mobile phases with 0.1% formic acid solution in water (A) and 0.1% formic acid and acetonitrile solution (B) were used. The flow rate of the mobile phase was maintained at 0.5 ml/min. The extract solution was prepared by mixing, and 1.0 μl in HPLC grade methanol was injected for 25 min and post-run time was 5 min. The gas used as the nebulizing source was nitrogen at the flow rate of 25 and 600 L/hour and drying gas was also used. The temperature optimized for analysis was 350°C. The capillary voltage used for analysis was 3,500 V, and fragmentation voltage was maintained at 125 V (Saleem et al., 2019; Saleem et al., 2020). The data were further processed with Agilent Mass Hunter, and the tentative qualitative identification of phytochemicals was achieved by METLIN database library search.

### Antioxidant Assays

The antioxidant methods as described earlier by Grochowski et al. (2019) were utilized to estimate the radical scavenging activity (DPPH and ABTS), reduction power (FRAP and CUPRAC), total antioxidant capacity (phosphomolybdenum assay), and metal chelation activity of the *C. gigantea* leaves ethanolic extract. The results of all the antioxidant assays were reported as milligrams of Trolox equivalents per gram of extract (mg TE/g extract), while metal chelating activity was expressed as milligrams of ethylenediaminetetraacetic acid equivalent (mg EDTAE/g extract) per gram of extract.

### Enzyme Inhibition Assays

*C. gigantea* (L.) Dryand leaves ethanolic extract was tested for its enzyme inhibition potential against AChE, BChE, tyrosinase, and α-amylase enzymes utilizing standard *in vitro* methods, as mentioned earlier (Mollica et al., 2017). Galantamine was the standard used for AChE and BChE, and inhibitory action was measured as mg GALAE/g extract (milligrams of galantamine equivalent per gram of extract). Likewise, acarbose was used as a standard for inhibition of the α-amylase enzyme, and the findings were reported in millimoles of acarbose equivalent per gram of extract (mmol ACAE/g extract). The effects of the inhibition of the tyrosinase enzyme were reported as milligrams of kojic acid equivalents per gram of extract (mg KAE/g extract) using kojic acid as a standard.

### *In Vivo* Wound Healing Activity

#### Ointment Preparation

The extract ointment was formulated in a simple ointment, namely, with two different extract concentrations, 0.5 percent (w/w) and 2.0 percent (w/w), respectively. The simple ointment containing wool fat (5 gm), hard paraffin (5 gm), cetostearyl alcohol (5 gm), and white soft paraffin (85 gm) was prepared as per Gemeda et al. (2018); (Gemeda et al., 2018). Nitrofurazone ointment (0.2 percent w/w) was used as a reference drug to compare the extract wound healing ability in the various animal models.

#### Experimental Design

Albino Wister male rats weighing 180–200 g were divided into four groups, each consisting of 6 rats following the previously established protocols (Harish et al., 2008):

Group 1: Control group treated with simple ointment

Group 2: Standard group treated with 0.2 percent nitrofurazone

Group 3: Test group 1 treated with 0.5 percent w/w ECGLD (0.5 g extract in simple, 100 g ointment)

Group 4: Test group 2 treated with 2 percent w/w ECGHD (2 g extract in simple, 100 g ointment)

#### Excision Wound Model

The rats were depilated on the back, and an area of 500 mm^2^ of maximum skin thickness was excised in the interscapular dorsal region as defined by Morton and Malone (1972). The ointment was applied topically every day until the day of the procedure, and complete epithelialization was achieved. On the 4^th^, 8^th^, 12^th^, and 16^th^ days, wound closure was assessed to see the percentage of wound closure and epithelization, indicating the development of new epithelial tissue to cover the wound. Epithelization time is the number of days needed to minimize the scar without having traces of the raw wound (Balakrishnan et al., 2006). Percent wound contraction was calculated by the following formula:

$$\text{Percent wound contraction}=\left[{\text{A}}_{0}-{\text{A}}_{\text{n}}\right]/{\text{A}}_{0}\times 100,$$

where A_0_ and A_n_ are initial wound area and wound area after “n” days, where n is day 4, day 8, day 12, and day 16.

#### Burn Wound Model

In overnight fasting animals under anesthesia with pentobarbitone (30 mg/kg IP), partial-thickness burn wounds were inflicted by pouring 80°C hot molten wax. The wax was poured on the animal shaved back through a circular opening nozzle of 300 mm^2^. The wax had been allowed to stay on the skin until it solidified. The ointment or vehicle was applied topically every day on the injury and wound contraction was measured as described on the following days (Rashed et al., 2003).

$$\text{Percent wound healing}=\left[{\text{A}}_{0}-{\text{A}}_{\text{n}}\right]/{\text{A}}_{0}\times 100,$$

where A_0_ and A_n_ are initial wound area and wound area after “n” days, where n is day 4, day 8, day 12, and day 16.

### Statistical Analysis

All results were the mean of three equivalent experiments and were expressed as average ±SD of value. The values are expressed as the mean ± SEM for animal models. Unpaired *t*-tests were used to assess the *p* value and significance compared to the control group with Graph Pad Prism version 6.04 for Windows.

## Results and Discussion

### Phytochemical Composition

**FIGURE 1:**
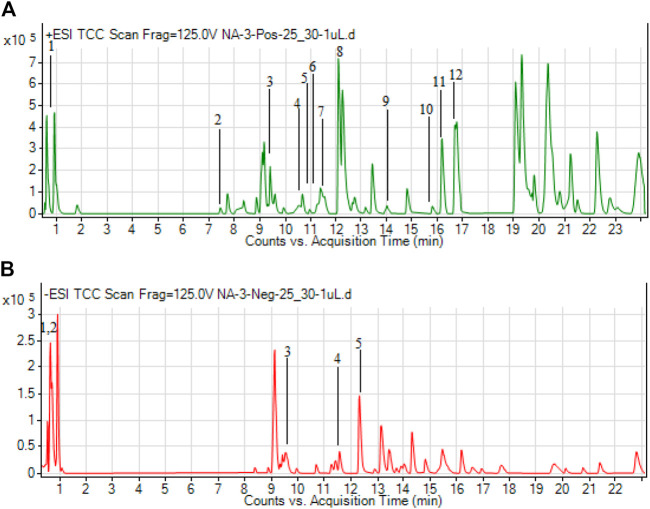
Total ion chromatograms (TICs) of ethanolic extract of *C. gigantea* (L.) Dryand leaves ethanolic in positive **(A)** and negative **(B)** ionization modes.

In the present study, the total bioactive content of *C. gigantea* (L.) Dryand leaves ethanolic extract was measured by evaluating total phenolic and total flavonoid contents. As presented in Table 1, the tested extract was found to have higher total flavonoid contents (46.75 mg RE/g extract) than phenolic contents (33.71 mg GAE/g extract). Similarly, to gain more insight into the chemical composition, the secondary metabolites profiling of *C. gigantea* (L.) Dryand leaves ethanolic was carried out by UHPLC-MS in positive and negative ionization modes. The tentatively identified compounds (as per METLIN database) are listed in Tables 2, 3, respectively, while their total ion chromatograms (TICs) are presented in Figures 1A, B. As seen in Table 2, the UHPLC-MS chromatograms base peak analysis tentatively identified 12 and 5 distinct phytocompounds in positive and negative ionization modes, respectively.

**TABLE 1:** Total bioactive contents, antioxidant, and enzyme inhibition activities of ethanolic extract of *C. gigantea* (L.) Dryand leaves.

*C. gigantea* (L.) Dryand a leaves ethanolic extract	
Total bioactive contents	
Total phenolic content (mg GAE/g extract)	33.71±0.13
Total flavonoid content (mg RE/g extract)	46.75±1.30

Antioxidant assays	
DPPH (mg TE/g extract)	67.90±0.75
ABTS (mg TE/g extract)	89.67±2.06
FRAP (mg TE/g extract)	118.8±0.35
CUPRAC (mg TE/g extract)	207.29±1.18
Phosphomolybdenum (mg TE/g extract)	1.71±0.05
Metal chelating (mg EDTAE/g extract)	27.74±0.63

Enzyme inhibition assays	
AChE (mg GALAE/g extract)	4.63±0.26
BChE (mg GALAE/g extract)	5.78±0.62
α-Amylase (mmol ACAE/g extract)	0.66±0.01
Tyrosinase (mg KAE/g extract)	81.72±0.36

Data from three repetitions with a mean ± standard deviation. GAE, gallic acid equivalent; RE, rutin equivalent.

**TABLE 2:** UHPLC-MS analysis of the ethanolic extract of *C. gigantea* (L.) Dryand leaves (positive ionization mode).

S.No	RT (min)	B. peak (*m/z*)	Proposed compound	Compound class	Mol. Formula	Mol. Mass
1	0.708	272.10	Deidaclin	Glucoside	C_12_H_17_NO_6_	271.10
2	7.46	422.21	Armillane	Sesquiterpene	C_23_H_32_O_7_	420.21
3	9.327	479.11	Tamarixin	Flavonoid	C_22_H_22_O_12_	478.11
4	10.693	212.15	Elaeokanine C	Alkaloid	C_12_H_21_NO_2_	211.15
5	10.975	247.19	Isoavocadienofuran	Furan deriv	C_17_H_26_O	246.19
6	11.264	227.15	Dihydrojasmonic acid, methyl ester	Jasmonic acid deriv	C_13_H_22_O_3_	226.15
7	11.575	317.05	Nodifloretin	Flavonoid	C_16_H_12_O_7_	316.05
8	12.263	273.23	2-Hydroxyhexadecanoic acid	Fatty acid	C_16_H_32_O_3_	272.23
9	14.023	295.18	Gingerol	Phenol	C_17_H_26_O_4_	294.18
10	15.814	235.16	Curcumenol	Sesquiterpenoid	C_15_H_22_O_2_	234.16
11	16.185	299.25	*cis*-9,10-Epoxystearic acid	Fatty acid	C_18_H_34_O_3_	298.25
12	16.73	279.15	Emmotin A	Sesquiterpenoid	C_16_H_22_O_4_	278.15

RT, retention time; B. peak: base peak.

**TABLE 3:** UHPLC-MS analysis of the ethanolic extract of *C. gigantea* leaves (negative ionization mode).

S.No	RT (min)	B. peak (*m/z*)	Proposed compound	Compound class	Mol. Formula	Mol. Mass
1	0.627	215.04	Isobergaptene	Coumarin	C_12_H_8_O_4_	216.04
2	0.658	341.12	Dictyoquinazol C	Alkaloid	C_18_H_18_N_2_O_5_	342.12
3	9.533	187.10	Nonic acid	Terpene	C_9_H_16_O_4_	188.10
4	11.449	329.24	5,8,12-Trihydroxy-9-octadecenoic acid	Fatty acid	C_18_H_34_O_5_	330.24
5	12.329	221.12	(6S)-Dehydrovomifoliol	Sesquiterpenoid	C_13_H_18_O_3_	222.12

RT, retention time; B. peak: base peak.

### Antioxidant Activity

Related antioxidant protocols have been performed to investigate the antioxidant ability of studied plant extract, including radical scavenging activity (DPPH and ABTS), accompanied by antioxidant reduction (FRAP and CUPRAC), phosphomolybdenum, and metal chelation assays to confirm and evaluate antioxidant findings (Table 1). The results highlighted the potential of the tested extract as considerable DPPH (67.90 mg TE/g extract) and ABTS (89.67 TE/g extract) radical scavenger (Table 1). Similarly, to measure the reduction potential of the studied plant extract, reducing power assays, i.e., FRAP and CUPRAC methods, have been used, and the results are shown in Table 1. The tested extract exhibited the FRAP and CUPRAC reducing potential with values of 118.8 and 207.29 mg TE/g extract, respectively. Likewise, the tested extract capacity for phosphomolybdenum assay is depicted in Table 1, and presented the total antioxidant capacity with a value of 1.71 mg TE/g extract. Moreover, the chelating capacity of the extract was evaluated by the metal chelation assay, and the extract was found to be considerably active (27.74 mg EDTAE/g extract) (Table 1).

### Enzyme Inhibition Assays

In the current study, *C. gigantea* (L.) Dryand leaves ethanolic extract was screened against AChE, BChE, α-amylase, and tyrosinase, and the findings are reported in Table 1. The tested plant extract exhibited higher AChE inhibition (4.63 mg GALAE/g extract). The observed reduction of AChE can be due to phenolic behavior, and this evidence is confirmed as previous studies validate similar associations (Hasnat et al., 2013; Bilal et al., 2016). For the BChE inhibition, the extract was found to present the inhibition values of 5.78 mg GALAE/g extract (Table 1). As for α-amylase enzyme inhibition, the tested extract was found to exhibit weak inhibitory potential with the value of 0.66 mmol ACAE/g extract, while, in the case of tyrosinase enzyme, significant inhibitory activity was noted, i.e., 81.72 mg KAE/g extract (Table 1).

### Wound Healing Activity

#### Excision Wound Model

**FIGURE 2:**
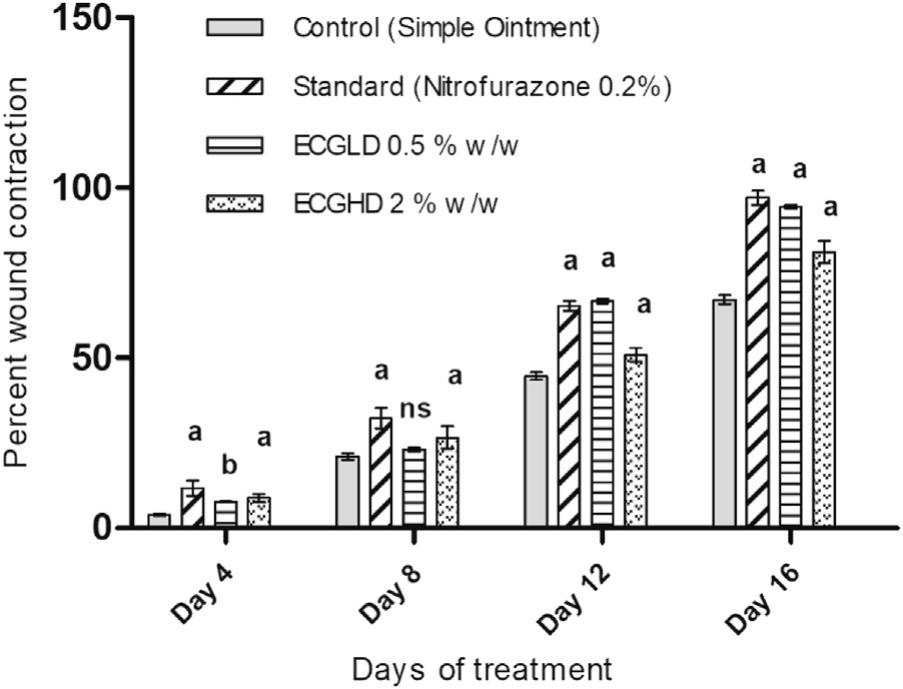
Percentage of wound contraction in excision wound model. ECPLD: extract of *C. gigantea* (L.) Dryand low dose; ECPHD: extract of *C. gigantea* (L.) Dryand high dose; a = *P < 0.001* vs. control; b = *P < 0.01* vs. control; c = *p < 0.05* vs. control.

**FIGURE 3:**
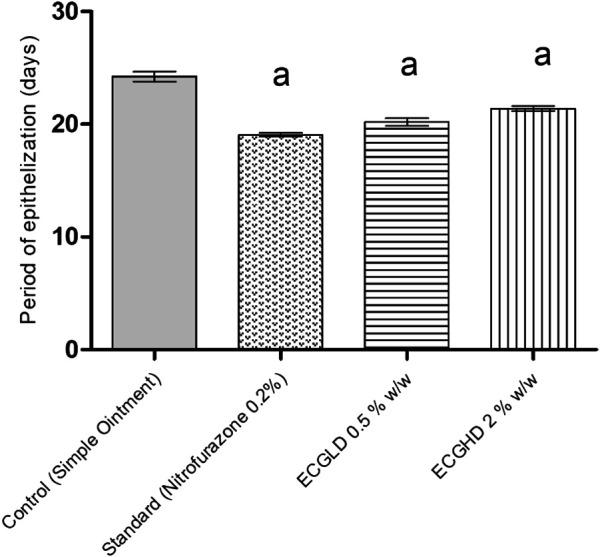
Period of epithelization in excision wound model. ECPLD: extract of *C. gigantea* (L.) Dryand low dose; ECPHD: extract of *C. gigantea* (L.) Dryand high dose; a = *P < 0.001* vs. control; b = *P < 0.01* vs. control; c = *p < 0.05* vs. control.

**FIGURE 4:**
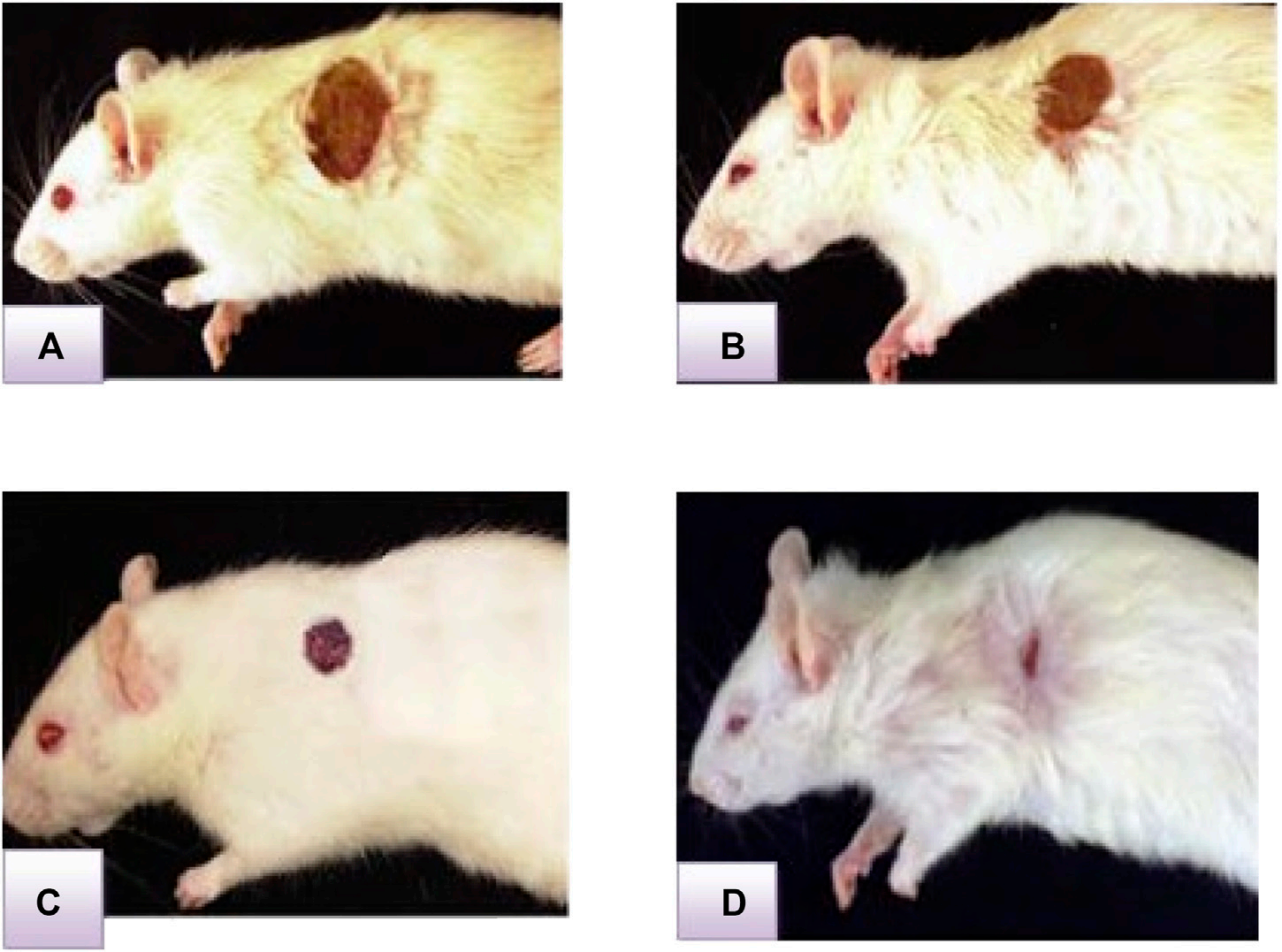
Representative image of the wound healing process in the excision wound model at ECGHD 2% w/w on the 4^th^ **(A)**, 8^th^ **(B)**, 12^th^ **(C)**, and 16^th^ **(D)** days of treatment.

The effects of wound healing as noted by the excision wound model are given in Table 4 and Figures 2, 3, 4, which represent 4, 8, 12, and 16 days of wound healing percentage for control (group treated with simple ointment), standard (nitrofurazone 0.2 percent w/w–treated group) and the test groups, namely, the ECPLD (*C. gigantea* (L.) Dryand extract low dose 0.5 percent w/w) and ECPHD (*C. gigantea* (L.) Dryand extract high dose 2 percent w/w). It was found that the capacity of the extract ointment to contract wound in both concentrations was significantly greater than that of the control (i.e., group treated with simple ointment). The wound contracting potential of animals treated with an ointment containing 0.5 percent (w/w) ethanolic extract on day 16 (94.33 ± 0.43) was found to be highly significant (*p < 0.001*) compared to the control group. The 2 percent (w/w) ointment-treated groups showed significant wound healing from the fourth day onwards, and the capacity to contract the wound was found to be significant (*p < 0.001*) on the 12^th^ and 16^th^ days (50.83 ± 1.94, 81.00 ± 3.26, respectively). Compared to control, both ECGLD (0.5 percent w/w) and ECGHD (2 percent w/w) provided a substantial decrease in the epithelization period. ECGLD therapy has achieved a significant decrease (*p < 0.001*) in epithelization time (20.20 ± 0.82). Some of the essential phytochemicals that have been reported for wound healing activity are related to flavonoids (Iyyam Pillai et al., 2010). Secondary metabolite analysis shows the existence of flavonoids (tamarixin) that may be accountable for their role in healing wounds.

**TABLE 4:** Effect of topical application of an ointment containing ethanolic extract of *C. gigantea* leaves on wound contraction of excision wound. Mean percentage of wound contraction ±SEM.

Groups	Treatment	Day 4	Day 8	Day 12	Day 16	Period of epithelization
Group 1	Control (simple ointment)	3.88 ± 0.20	21.02 ± 1.05	44.71 ± 1.12	67.12 ± 1.34	24.23 ± 1.07
Group 2	Standard (nitrofurazone) 0.2% w/w	11.68 ± 2.21***	32.23 ± 3.10 ***	65.28 ± 1.49***	97.05 ± 2.06***	19.07 ± 0.41***
Group 3	ECGLD 0.5% w/w	7.7 ± 0.16**	23.08 ± 0.62 ns	66.67 ± 0.65***	94.33 ± 0.43***	20.20 ± 0.82***
Group 4	ECGHD 2% w/w	8.800 ± 1.20***	26.62 ± 3.33***	50.83 ± 1.94***	81.00 ± 3.26***	21.40 ± 0.55***

ECGLD: extract of *C. gigantea* low dose; ECGHD: extract of *C. gigantea* high dose high dose. *n* = 6 animals in each group. The treated groups are analyzed by two-way ANOVA for wound closure and one-way ANOVA for the period of epithelization by comparing with the control group. **p < 0.01, ***p < 0.001, ns: not significant.

#### Burn Wound Model

**FIGURE 5:**
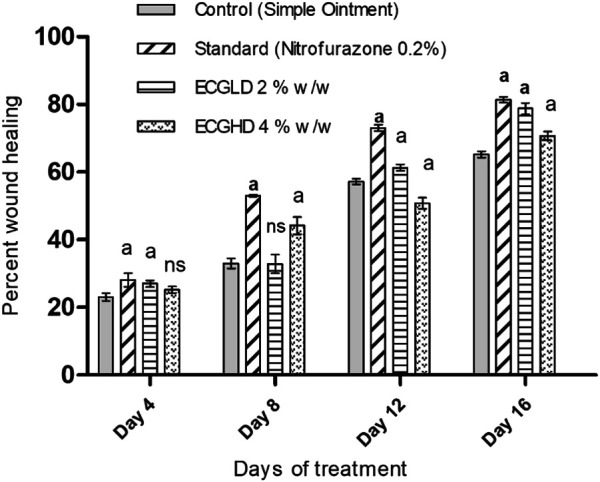
ECPLD: extract of *C. gigantea* (L.) Dryand low dose; ECPHD: extract of *C. gigantea* (L.) Dryand high dose; a = *P ≤ 0.001* vs. control; b = *p ≤ 0.01* vs. control; c = *p ≤ 0.05* vs. control.

**FIGURE 6:**
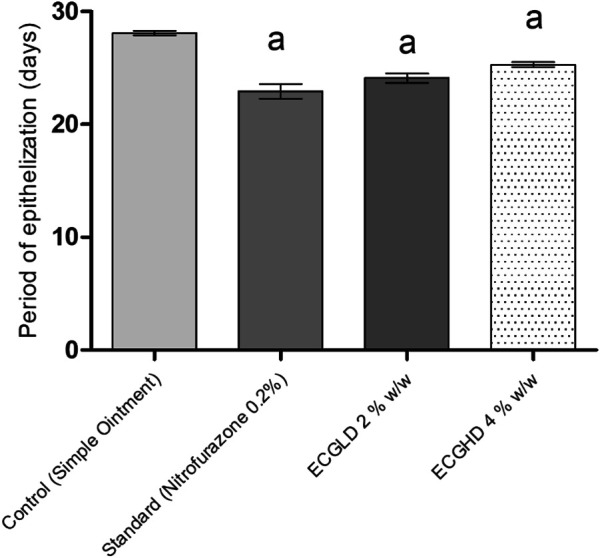
Period of epithelization in burn wound model. ECPLD: extract of *C. gigantea* (L.) Dryand low dose; ECPHD: extract of *C. gigantea* (L.) Dryand high dose; a = *P < 0.001* vs. control; b = *P < 0.01* vs. control; c = *P < 0.05* vs. control.

**FIGURE 7:**
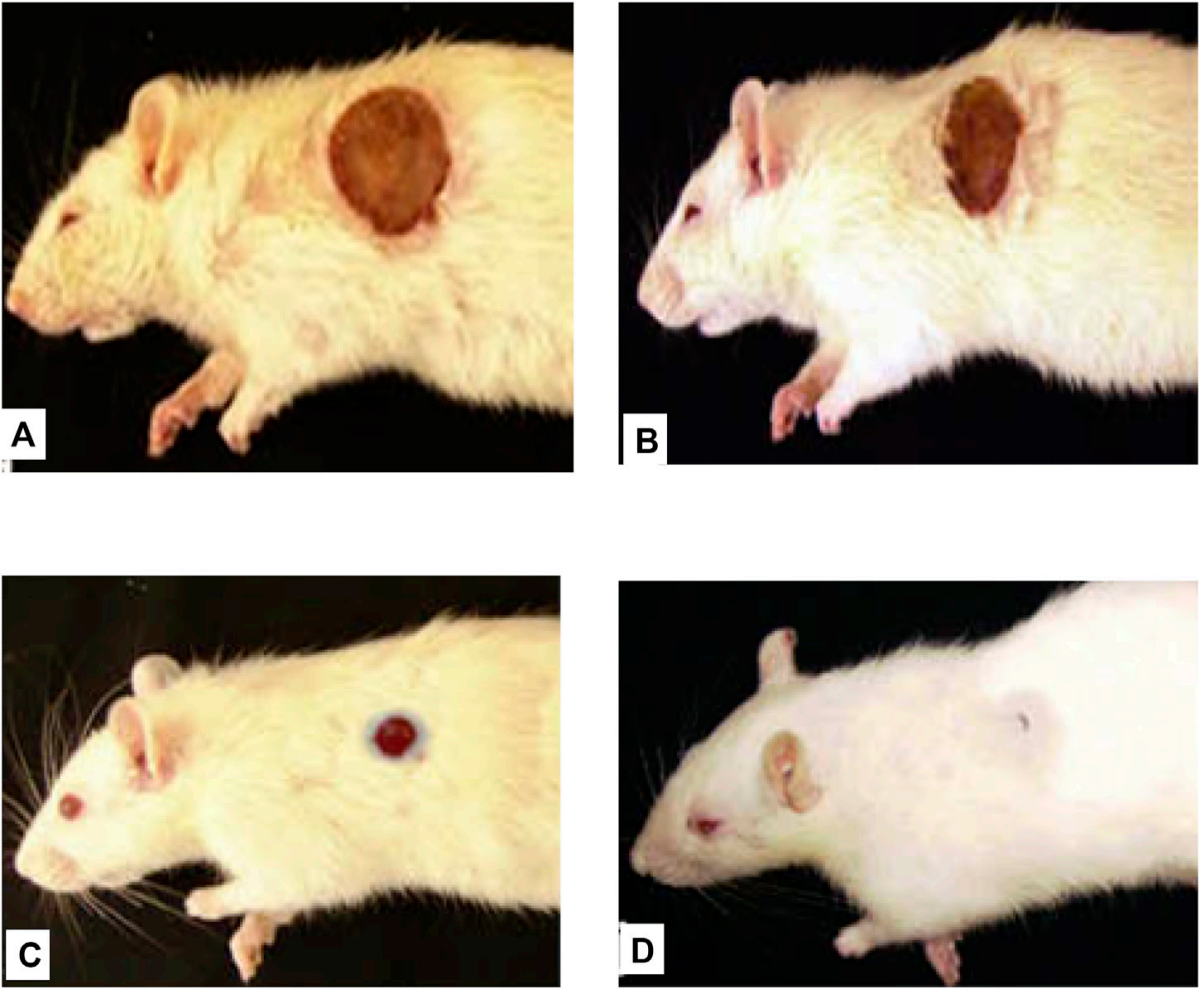
Representative image of the wound healing process in burn wound model at ECGHD 2% w/w on the 4^th^ **(A)**, 8^th^ **(B)**, 12^th^ **(C)**, and 16^th^ **(D)** days of treatment.

The results of burn wound healing activity are depicted in Table 5 and Figures 5, 6, 7, showing a percentage of wound healing at 4, 8, 12, and 16 days for control (simple ointment-treated group), standard (nitrofurazone, 0.2 percent w/w–treated group), and test groups, namely, the ECGLD (0.5 percent w/w) and ECGHD (2 percent w/w). Substantial improvement has been observed in percentage contractibility in ECGLD-treated rats from day 4 onwards, and even in later days, the closure rate is much higher than that in control groups. In contrast to the control group, the wound contracting capacity of animals treated with an ointment containing (0.5 percent w/w) ethanol extract was observed to be significantly higher (*p < 0.001*) on the 16^th^ day (78.75 ± 1.74). On the 16^th^ day (70.67 ± 1.32), the percentage of wound contraction in ECGHD 2 percent w/w–treated animals demonstrated moderate activity (*p 0.05*). A stronger healing trend and decrease in the epithelization period were found in ECGLD 0.5 percent w/w–treated group with extremely significant activity (*p < 0.001*) (24.10 ± 1.01) and ECGHD 2 percent w/w showed modest significant activity (*p < 0.01*) (25.30 ± 0.54) compared with the control group. In our study, flavonoids and terpenoids have been identified to have astringent and antimicrobial properties that promote the healing and epithelization of wounds (Iyyam Pillai et al., 2010). It is known that flavonoids also inhibit lipid peroxidation not only through blocking or minimizing cell necrosis but also by enhancing vascularity and improving the viability of collagen fibrils (Getie et al., 2002).

**TABLE 5:** Effect of topical application of an ointment containing ethanolic leaf extract of *C. gigantea* leaves on wound contraction of the burn wound. Mean percentage of wound contraction ±SEM.

Groups	Treatment	4th day	8th day	12th day	16th day	Period of epithelization
Group 1	Control (simple ointment)	23.09 ± 1.15	33.00 ± 1.45	57.19 ± 0.83	65.18 ± 0.92	28.09 ± 0.47
Group 2	Standard (nitrofurazone) 0.2% w/w	28.12 ± 2.03***	53.02 ± 0.32***	73.07 ± 0.95***	81.38 ± 0.81***	22.93 ± 1.57***
Group 3	ECGLD 2% w/w	27.00 ± 0.95***	32.83 ± 2.76 ns	61.33 ± 0.99***	78.75 ± 1.71***	24.10 ± 1.00***
Group 4	ECGHD 4% w/w	25.25 ± 0.95 ns	44.17 ± 2.55***	50.78 ± 1.70***	70.67 ± 1.31***	25.30 ± 0.54***

ECGLD: extract of *C. gigantea* low dose; ECGHD: extract of *C. gigantea* high dose. *n* = 6 animals in each group. The treated groups are analyzed by two-way ANOVA for wound closure and -way ANOVA for the period of epithelization by comparing with the control group. ***p < 0.001, ns: not significant.

## Discussion

Plants bioactive compounds, including phenolic and flavonoids, have several beneficial effects on human health (Dirir et al., 2017). Mostly these compounds were found to be derivatives of sesquiterpene, flavonoids, alkaloids, fatty acids, and phenols. The two identified sesquiterpenes (armillane and emmotin A), flavonoids (tamarixin), alkaloids (elaeokanine C), phytoconstituents (isoavocadienofuran, dihydrojasmonic acid, methyl ester, nodifloretin, curcumenol, isobergapten, dictyoquinazol C (6S)-dehydrovomifoliol), fatty acid (2-hydroxyhexadecanoic acid, *cis*-9,10-epoxystearic acid, 5,8,12-trihydroxy-9-octadecenoic acid), terpene (nonic acid), phenol (gingerol), glucoside (deidaclin). The presence of these phytochemical classes, specifically alkaloids, phenolics, terpenes, flavonoids, is in accordance with the previous studies that have reported the presence of these classes as major phytochemical groups in this plant species (Kumar et al., 2011).

Free radicals such as DPPH and ABTS are widely used to test various plant samples for their radical scavenging ability (Sarıkürkçü et al., 2017). The former higher content of total phenolic and flavonoids may be ascribed and associated with this observed higher antioxidant potential of these extracts. Interestingly, the increased antioxidant function of methanol extracts was also illustrated by previous researchers (Li et al., 2017; Sumczynski et al., 2017). Previously, the antioxidant effects of the methanolic, chloroform, hot water, and ethyl acetate extracts of the leaves of *C. gigantea* (L.) Dryand were *via* DPPH, reducing power, and inhibition of nitric oxide radical, and the chloroform extracts showed the highest antioxidant activity (Mohanraj and Usmani, 2012). Total antioxidant activity was calculated using phosphomolybdenum assays based on molybdenum (VI) reduction to molybdenum (V) compounds with antioxidant potential and the subsequent development of molybdenum (V) complex (green in color) (Prieto et al., 1999). The phenolic and flavonoid content could be related to the observed antioxidant ability of the tested extract, and our findings are consistent with several previous studies suggesting that the amount of bioactive content in plant extracts plays an important role and accounts for the capacity of antioxidants (Patro et al., 2016; Nordin et al., 2018). Therefore, our findings could be regarded as the results of a preliminary investigation to analyze further the use of *C. gigantea* (L.) Dryand as a possible source of natural antioxidant molecules.

In recent years, enzymes have been studied as an important research area to prevent common health-related issues worldwide (Rauf and Jehan, 2017). AChE and BChE (cholinesterases) enzyme inhibitors are presently being utilized for the treatment/management of Alzheimer’s diseases (AD). Likewise, α-amylase is an enzyme responsible for releasing glucose from starch and is also involved in the carbohydrates catabolism (Kubínova et al., 2014). Consequently, the compounds with inhibition potential against α-amylase and cholinesterases could be considered key molecules for treating the diseases linked with these enzymes (Yerlikaya et al., 2017). Tyrosinase is a crucial enzyme in melanin synthesis, so it can be used as the primary target for controlling hyperpigmentation problems (Pillaiyar et al., 2017). Tacrine, acarbose, and kojic acid are some of the available compounds that have been used as inhibitors for cholinesterases, glucosidases, and tyrosinase, respectively. However, these commercially available inhibitors have reported adverse effects (Mishra et al., 2019; Milovanovic et al., 2021). Henceforward, the search for new enzyme inhibitors from natural flora may therefore be an appropriate alternative to overcome the above-mentioned deficiencies.

## Conclusion

In this study, the phytochemical profile, antioxidants, enzyme inhibition effects, and secondary metabolites profile of *C. gigantea* (L.) Dryand leaves ethanolic extract are explored. The plant was found to have a substantial phenolic and flavonoid content. As identified by the UHPLC-MS study, the secondary metabolite profile indicated the existence of several bioactive metabolites belonging to important phytochemical groups. Overall, the plant extract under study was found to have notable antioxidant and enzyme inhibitory ability, which could be attributed to its total bioactive and secondary metabolite composition. Thus, this plant should be viewed as a perspective origin for bioactive phytocompounds having antioxidant and enzyme inhibition potential. In addition, the current research justifies the use of *C. gigantea* (L.) Dryand leaves to facilitate wound healing in the traditional medicinal system. However, further studies on the isolation of responsible lead compounds and their toxicity are highly recommended.

## Data Availability

The raw data supporting the conclusions of this article will be made available by the authors without undue reservation.

## References

[B1] Ahmad S. Abdel-Salam N. M. Ullah R. (2016). *In Vitro* antimicrobial Bioassays, DPPH Radical Scavenging Activity, and FTIR Spectroscopy Analysis of Heliotropium Bacciferum. Biomed. Res. Int. 2016, 3818945. 10.1155/2016/3818945 27597961 PMC4997099

[B2] Argal A. Pathak A. K. (2006). CNS Activity of Calotropis Gigantea Roots. J. Ethnopharmacology 106, 142–145. 10.1016/j.jep.2005.12.024 16446065

[B3] Aumeeruddy M. Z. Mahomoodally M. F. (2019). Combating Breast Cancer Using Combination Therapy with 3 Phytochemicals: Piperine, Sulforaphane, and Thymoquinone. Cancer 125, 1600–1611. 10.1002/cncr.32022 30811596

[B4] Balakrishnan B. Mohanty M. Fernandez A. C. Mohanan P. V. Jayakrishnan A. (2006). Evaluation of the Effect of Incorporation of Dibutyryl Cyclic Adenosine Monophosphate in an In Situ-forming Hydrogel Wound Dressing Based on Oxidized Alginate and Gelatin. Biomaterials 27, 1355–1361. 10.1016/j.biomaterials.2005.08.021 16146648

[B5] Bilal S. Khan A. Waqas M. Shahzad R. Kim I.-D. Lee I.-J. (2016). Biochemical Constituents and *In Vitro* Antioxidant and Anticholinesterase Potential of Seeds from Native Korean Persimmon Genotypes. Molecules 21, 893. 10.3390/molecules21070893 PMC6274387 27399664

[B6] Chew Y.-L. Goh J.-K. Lim Y.-Y. (2009). Assessment of *In Vitro* Antioxidant Capacity and Polyphenolic Composition of Selected Medicinal Herbs from Leguminosae Family in Peninsular Malaysia. Food Chem. 116, 13–18. 10.1016/j.foodchem.2009.01.091

[B7] Cuthbertson D. J. Johnson S. R. Piljac-Žegarac J. Kappel J. Schäfer S. Wüst M. (2013). Accurate Mass-Time Tag Library for LC/MS-based Metabolite Profiling of Medicinal Plants. Phytochemistry 91, 187–197. 10.1016/j.phytochem.2013.02.018 23597491 PMC3697863

[B8] Dirir A. M. Cheruth A. J. Ksiksi T. S. (2017). Ethnomedicine, Phytochemistry and Pharmacology of Calotropis Procera and Tribulus Terrestris. J. Nat. Remedies 17 (2), 38–47. 10.18311/jnr/2017/11043

[B9] Garber J. C. Barbee R. W. Bielitzki J. T. Clayton L. Donovan J. Hendriksen C. (2011). Guide for the Care and Use of Laboratory Animals, 8. Washington DC: The National Academic Press, 220.

[B10] Gemeda N. Tadele A. Lemma H. Girma B. Addis G. Tesfaye B. (2018). Development, Characterization, and Evaluation of Novel Broad-Spectrum Antimicrobial Topical Formulations from Cymbopogon Martini (Roxb.) W. Watson Essential Oil. Evidence-Based Complement. Altern. Med. 2018, 1–16. 10.1155/2018/9812093 PMC6151366 30275867

[B11] Getie M. Gebre-Mariam T. Rietz R. Neubert R. H. (2002). Evaluation of the Release Profiles of Flavonoids from Topical Formulations of the Crude Extract of the Leaves of Dodonea Viscosa (Sapindaceae). Pharmazie 57, 320–322. 12061256

[B12] Ghule S. D. Vidyasagar G. Bhandari A. Sharma P. Gunjal A. P. (2014). CNS Activity of Leaves Extract of Calotropis Gigantea. Asian Pac. J. Trop. Dis. 4, S902–S905. 10.1016/s2222-1808(14)60755-6

[B13] Grochowski D. M. Uysal S. Zengin G. Tomczyk M. (2019). *In Vitro* antioxidant and Enzyme Inhibitory Properties of Rubus Caesius L. Int. J. Environ. Health Res. 29, 237–245. 10.1080/09603123.2018.1533532 30311781

[B14] Harish B. G. Krishna V. Santosh Kumar H. S. Khadeer Ahamed B. M. Sharath R. Kumara Swamy H. M. (2008). Wound Healing Activity and Docking of Glycogen-Synthase-Kinase-3-β-Protein with Isolated Triterpenoid Lupeol in Rats. Phytomedicine 15, 763–767. 10.1016/j.phymed.2007.11.017 18222664

[B15] Hasnat M. Pervin M. Lim B. (2013). Acetylcholinesterase Inhibition and *In Vitro* and *In Vivo* Antioxidant Activities of Ganoderma Lucidum Grown on Germinated Brown rice. Molecules 18, 6663–6678. 10.3390/molecules18066663 23749158 PMC6269759

[B16] Iyyam Pillai S. Palsamy P. Subramanian S. Kandaswamy M. (2010). Wound Healing Properties of Indian Propolis Studied on Excision Wound-Induced Rats. Pharm. Biol. 48, 1198–1206. 10.3109/13880200903578754 20819020

[B17] Kähkönen M. P. Hopia A. I. Vuorela H. J. Rauha J.-P. Pihlaja K. Kujala T. S. (1999). Antioxidant Activity of Plant Extracts Containing Phenolic Compounds. J. Agric. Food Chem. 47, 3954–3962. 10.1021/jf990146l 10552749

[B18] Khurm M. Chaudhry B. Uzair M. Janbaz K. (2016). Antimicrobial, Cytotoxic, Phytotoxic and Antioxidant Potential of Heliotropium Strigosum Willd. Medicines 3, 20. 10.3390/medicines3030020 PMC5456253 28930129

[B19] Kubínová R. Pořízková R. Navrátilová A. Farsa O. Hanáková Z. Bačinská A. (2014). Antimicrobial and Enzyme Inhibitory Activities of the Constituents of Plectranthus Madagascariensis (Pers.) Benth. J. Enzyme Inhib. Med. Chem. 29, 749–752. 10.3109/14756366.2013.848204 24506206

[B20] Kumar G. Karthik L. Rao K. V. B. (2011). A Review on Pharmacological and Phytochemical Profile of Calotropis Gigantea Linn. Pharmacologyonline 1, 1–8.

[B21] Li H. Zhang D. Tan L.-H. Yu B. Zhao S.-P. Cao W.-G. (2017). Comparison of the Antioxidant Properties of Various Solvent Extracts from Dipsacus Asperoides and Identification of Phenolic Compounds by LC-ESI-QTOF-MS-MS. South Afr. J. Bot. 109, 1–8. 10.1016/j.sajb.2016.12.018

[B22] Mahomoodally M. Aumeeruddy M. Rengasamy K. R. Roshan S. Hammad S. Pandohee J. (2019). Ginger and its Active Compounds in Cancer Therapy: From Folk Uses to Nano-Therapeutic Applications. Semin. Cancer Biol. 69, 140–149. 10.1016/j.semcancer.2019.08.009 31412298

[B23] Milovanovic I. Zengin G. Maksimovic S. Tadic V. (2021). Supercritical and Ultrasound‐assisted Extracts from Pleurotus Pulmonarius Mushroom: Chemical Profiles, Antioxidative and Enzyme Inhibitory Properties. J. Sci. Food Agric. 101 (6), 2284–2293. 10.1002/jsfa.10849 33006768

[B24] Mishra P. Kumar A. Panda G. (2019). Anti-cholinesterase Hybrids as Multi-Target-Directed Ligands against Alzheimer's Disease (1998-2018). Bioorg. Med. Chem. 27, 895–930. 10.1016/j.bmc.2019.01.025 30744931

[B25] Mohanraj R. Usmani M. A. (2012). Antioxidant Activity of the Leaf Extracts of Calotropis Procera. Int. J. Adv. Biotechnol. Res. 2, 47–52.

[B26] Mollica A. Zengin G. Locatelli M. Stefanucci A. Mocan A. Macedonio G. (2017). Anti-diabetic and Anti-hyperlipidemic Properties of Capparis Spinosa L.: *In Vivo* and *In Vitro* Evaluation of its Nutraceutical Potential. J. Funct. Foods 35, 32–42. 10.1016/j.jff.2017.05.001

[B27] Morton J. J. Malone M. H. (1972). Evaluation of Vulneray Activity by an Open Wound Procedure in Rats. Arch. Int. Pharmacodyn. Ther. 196, 117–126. 5059357

[B28] Nordin M. L. Kadir A. A. Zakaria Z. A. Abdullah R. Abdullah M. N. H. (2018). *In Vitro* investigation of Cytotoxic and Antioxidative Activities of Ardisia Crispa against Breast Cancer Cell Lines, MCF-7 and MDA-MB-231. BMC Complement. Altern. Med. 18, 87. 10.1186/s12906-018-2153-5 29530022 PMC5848562

[B29] Pathak A. K. Argal A. (2007). Analgesic Activity of Calotropis Gigantea Flower. Fitoterapia 78, 40–42. 10.1016/j.fitote.2006.09.023 17113726

[B30] Patro G. Bhattamisra S. Mohanty B. Sahoo H. (2016). *In Vitro* and *In Vivo* Antioxidant Evaluation and Estimation of Total Phenolic, Flavonoidal Content of Mimosa Pudica L. Phcog Res. 8, 22. 10.4103/0974-8490.171099 26941532 PMC4753756

[B31] Pillaiyar T. Manickam M. Namasivayam V. (2017). Skin Whitening Agents: Medicinal Chemistry Perspective of Tyrosinase Inhibitors. J. Enzyme Inhib. Med. Chem. 32, 403–425. 10.1080/14756366.2016.1256882 28097901 PMC6010116

[B32] Prieto P. Pineda M. Aguilar M. (1999). Spectrophotometric Quantitation of Antioxidant Capacity through the Formation of a Phosphomolybdenum Complex: Specific Application to the Determination of Vitamin E. Anal. Biochem. 269, 337–341. 10.1006/abio.1999.4019 10222007

[B33] Rashed A. Afifi F. Disi A. (2003). Simple Evaluation of the Wound Healing Activity of a Crude Extract of Portulaca Oleracea L. (Growing in Jordan) in *Mus musculus* JVI-1. J. Ethnopharmacology 88, 131–136. 10.1016/s0378-8741(03)00194-6 12963132

[B34] Rauf A. Jehan N. (2017). Natural Products as a Potential Enzyme Inhibitors from Medicinal Plants. Enzyme Inhibitors and Activators. Rijeka: InTech, 165–177.

[B35] Saleem H. Htar T. T. Naidu R. Nawawi N. S. Ahmad I. Ashraf M. (2019). Biological, Chemical and Toxicological Perspectives on Aerial and Roots of Filago Germanica (L.) Huds: Functional Approaches for Novel Phyto-Pharmaceuticals. Food Chem. Toxicol. 123, 363–373. 10.1016/j.fct.2018.11.016 30419323

[B36] Saleem H. Zengin G. Locatelli M. Tartaglia A. Ferrone V. Htar T. T. (2020). Filago Germanica (L.) Huds. Bioactive Constituents: Secondary Metabolites Fingerprinting and *In Vitro* Biological Assays. Ind. Crops Prod. 152, 112505. 10.1016/j.indcrop.2020.112505

[B37] Sarıkürkçü C. Cengiz M. Küçükyumru A. Zengin G. (2017). Determination of Antioxidant Activities of Solvent Extracts from an Endemic Plant: Phlomis Leucophracta. Marmara Pharm. 22 (1), 86–90. 10.12991/mpj.2018.45

[B38] Shanmugam S. Bhavani P. (2014). Studies on the Comparison of Phytochemical Constituents and Antimicrobial Activity of Curcuma Longa Varieties. Int. J. Curr. Microbiol. Appl. Sci. 3, 573–581.

[B39] Srivastava S. Singh A. Rawat A. (2015). Comparative Botanical and Phytochemical Evaluation of Calotropis Procera Linn. And Calotropis Gigantea Linn. Root. J. App Pharm. Sci. 5, 041–047. 10.7324/japs.2015.50707

[B40] Sumczynski D. Kotásková E. Orsavová J. Valášek P. (2017). Contribution of Individual Phenolics to Antioxidant Activity and *In Vitro* Digestibility of Wild Rices (Zizania Aquatica L.). Food Chem. 218, 107–115. 10.1016/j.foodchem.2016.09.060 27719885

[B41] Suroowan S. Mahomoodally M. F. (2019). Herbal Medicine of the 21st century: a Focus on the Chemistry, Pharmacokinetics and Toxicity of Five Widely Advocated Phytotherapies. Ctmc 19, 2718–2738. 10.2174/1568026619666191112121330 31721714

[B42] Yerlikaya S. Zengin G. Mollica A. Baloglu M. C. Celik Altunoglu Y. Aktumsek A. (2017). A Multidirectional Perspective for Novel Functional Products: *In Vitro* Pharmacological Activities and In Silico Studies on Ononis natrix Subsp. Hispanica. Front. Pharmacol. 8, 600. 10.3389/fphar.2017.00600 28919860 PMC5585257

